# Persistent Food Insecurity and Material Hardships: A Latent Class Analysis of Experiences among Venezuelan Refugees and Migrants in Urban Colombia

**DOI:** 10.3390/nu16071060

**Published:** 2024-04-04

**Authors:** Andrea L. Wirtz, Megan Stevenson, José Rafael Guillén, Jennifer Ortiz, Miguel Ángel Barriga Talero, Kathleen R. Page, Jhon Jairo López, Jhon Fredy Ramirez Correa, Damary Martínez Porras, Ricardo Luque Núñez, Julián Alfredo Fernández-Niño, Paul B. Spiegel

**Affiliations:** 1 Department of Epidemiology, Center for Public Health and Human Rights, Johns Hopkins University Bloomberg School of Public Health, Baltimore, MD 21205, USA; msteve48@jhmi.edu (M.S.); kpage2@jhmi.edu (K.R.P.); 2 Department of International Health, Center for Humanitarian Health, Johns Hopkins University Bloomberg School of Public Health, Baltimore, MD 21205, USA; pbspiegel@jhu.edu; 3 Red Somos, Bogotá 111321, Colombia; jrguillen@gmail.com (J.R.G.); jenniferlorena.ortiz@alum.upf.edu (J.O.); mquimica2004@gmail.com (M.Á.B.T.); jholopeza@gmail.com (J.J.L.); prevencion_saludsexual@redsomos.org (J.F.R.C.); damarymartinez@gmail.com (D.M.P.); 4 Department of Infectious Disease, Johns Hopkins School of Medicine, Baltimore, MD 21205, USA; 5 Ministry of Health and Social Protection, Bogotá 110311, Colombia; rluque@minsalud.gov.co; 6 Departamento de Salud Pública, Universidad del Norte, Barranquilla 080003, Colombia; aninoj@uninorte.edu.co

**Keywords:** migrant, refugee, food security, material hardship, Colombia, Venezuela, violence, infectious disease

## Abstract

The causes and conditions of displacement often increase the vulnerability of migrant and refugee populations to food insecurity, alongside other material hardships. We aimed to examine the multidimensional aspects and patterns of food insecurity and other material hardships in a cross-sectional sample of 6221 Venezuelan refugees and migrants in urban Colombia using a latent class analysis. Using multinomial and logistic regression models, we investigated the demographic and migratory experiences associated with identified classes and how class membership is associated with multiple health outcomes among Venezuelan refugees and migrants, respectively. Approximately two thirds of the sample was comprised cisgender women, and the participants had a median age of 32 years (IQR: 26–41). Four heterogeneous classes of food insecurity and material hardships emerged: Class 1—low food insecurity and material hardship; Class 2—high food insecurity and material hardship; Class 3—high income hardship with insufficient food intake; and Class 4—income hardship with food affordability challenges. Class 2 reflected the most severe food insecurity and material hardships and had the highest class membership; Venezuelans with an irregular migration status were almost 1.5 times more likely to belong to this class. Food insecurity and material hardship class membership was independently associated with self-rated health, mental health symptoms, and recent violence victimization and marginally associated with infectious disease outcomes (laboratory-confirmed HIV and/or syphilis infection). Social safety nets, social protection, and other interventions that reduce and prevent material hardships and food insecurity among refugees and migrants, alongside the host community, may improve public health, support development, and reduce healthcare costs. In the long term, regularization and social policies for migrants aimed at enhancing refugees’ and migrants’ social and economic inclusion may contribute to improving food security in this population.

## 1. Introduction

The ongoing displacement of over 7.7 million Venezuelans as of November 2023 outside their home country represents one of the largest humanitarian emergencies globally [1]. At the same time, the global population of displaced people is growing rapidly and is expected to continue to do so, with more persons expected to be displaced by climate-related factors [2]. While hunger and famine have long been characteristic of crises and displacements, the impacts of climate change, increasing numbers of displaced people, and the prioritization of food security as a Sustainable Development Goal bring a renewed focus on the role of food insecurity in these populations [3,4,5].

In Venezuela, the ongoing economic and political crisis has resulted in declining food security, an increasing prevalence of acute malnutrition, rising pediatric hospital admissions with acute malnutrition, and clinician reports of increasing child deaths attributed to acute malnutrition in between 2014 and 2019 [6]. In the time since that last report, more recent studies in the literature have suggested that the infant mortality rate in Venezuela has continued to climb [7]. The National Survey of Living Conditions in Venezuela (ENCOVI) estimated that 89% of Venezuelan homes were experiencing food insecurity in 2020, and this figure declined but remained high at 78% in 2022 [8]. Growing household poverty is considered a major driver, as the 2015 ENCOVI report estimated that 87% of Venezuelan households do not earn enough income to buy sufficient food, an increase from the 80% that was reported in 2014. This same survey estimated that 34% of the population was recently impoverished, 28% lived in chronic extreme poverty, and 19% were in some form of poverty that was not classified as extreme, chronic, or recent [9].

Of the more than 7.7 million Venezuelans residing outside their home country as of November 2023, over 6.54 million are estimated to be residing in the Latin American and Caribbean region. Sharing its border with Venezuela, Colombia is the most significant receiving country in the region, with 2.88 million Venezuelans estimated to reside in Colombia [1]. Research on the Venezuelan refugees and migrants in other countries demonstrates ongoing food insecurity after relocation, with estimates varying from 39% to 91% in terms of food insecurity [10,11,12,13]. In prior reports, we estimated that only 7.9% of the Venezuelan refugees and migrants residing in four urban settings of Colombia were food secure in 2021–2022. The remainder were experiencing low (26.5%) or very low (65.7%) food security [14]. These reports also demonstrated significant material hardships [14,15]. However, estimates of food security—the ability of households or individuals to consistently access safe, culturally acceptable, and nutritious foods [16]—vary by measure. Further, summative indicators of food security fail to capture unique, heterogenous patterns and the co-occurrence of other material hardships, which are considered important predictors of a household or individual’s ability to meet basic needs [17].

This analysis aimed to examine the multidimensional aspects and patterns of food insecurity and other material hardships, broadly classified as expense hardship, food insecurity, and housing hardship [17], among Venezuelan refugees and migrants in Colombia through a latent class analysis. We further aimed to investigate the demographic and migratory experiences associated with identified classes. Because food insecurity and material hardships are recognized social determinants of health [18], we investigated how class membership is associated with multiple physical and mental health outcomes among Venezuelan refugees and migrants. Understanding patterns and relationships with these social determinants of health, food insecurity, and material hardships can inform policies and health and social protection programs for migrants and refugees in this context and in similar settings.

## 2. Materials and Methods

We used data from a cross-sectional survey implemented by Red Somos (Bogotá, Colombia), Johns Hopkins University (Baltimore, MD, USA), and the Colombian Ministry of Health and Social Protection (Bogotá, Colombia). The study activities were reviewed and approved by the Ethical Review Committee at Universidad el Bosque (Bogotá, Colombia) and the Institutional Review Board at Johns Hopkins School of Public Health (Baltimore, MD, USA). The protocol was also reviewed in accordance with the US Center for Disease Control and Prevention human research protection procedures. Methodological details and other results have been published previously [14,15,19,20].

*Study sample and setting:* We used respondent-driven sampling (RDS), a non-probability chain referral sampling method [21]. RDS is widely used across international settings to sample populations that lack a sampling frame. Our RDS began with 10 purposively selected ‘seeds’ from the target population, who participated in all study activities and were then asked to invite 4 adult Venezuelan peers (recruits) to participate in the study activities. Eligible and participating recruits were then asked to refer up to four more peer Venezuelans. This process continued until the target sample size was achieved [19].

The study was conducted in two conurbation sites of the following cities: (1) Bogotá and Soacha and (2) Barranquilla and Soledad. Candidate participants could be referred across and participate within any of the selected cities. The cities were chosen for their population distribution of Venezuelan refugees and migrants, available healthcare and humanitarian referral options for Venezuelans, and lower presence of Venezuelan refugees and migrants en route to another country. Data collection was conducted from 30 July 2021 to 5 February 2022 among 6221 participants.

Individuals were eligible to participate if they met the following criteria: aged 18 years or older, self-reported birth in Venezuela, self-reported migration to Colombia in 2015 or later, currently lived in a study city, did not report the intention to leave Colombia, and had a valid recruitment coupon. Enrollment was restricted to one member per immediate family. Eligible participants underwent initial screening, gave consent, and underwent literacy screening and data collection in a private study location. Those who consented completed a survey questionnaire and rapid HIV and syphilis testing with laboratory-based confirmatory testing. To limit in-person contact during COVID-19, participants with an appropriate literacy level completed a self-administered electronic questionnaire; participants with low literacy or those who reported discomfort with technology completed an interviewer-administered questionnaire.

*Survey measures:* The survey measures spanned the domains of demographics; migration status, motivation for migration or displacement, and displacement experience; food security; health history, including access to healthcare, chronic health conditions, self-rated health [22], and anxiety and depression, based on the 4-item Patient Health Questionnaire for Anxiety and Depression (PHQ4) [23]; experiences of violence and discrimination; and the use of humanitarian services. We included existing survey measures from the region that had been validated in migrant populations or in the Spanish language when possible [19]. Current migration status was measured as self-reported regular status, meaning that the individual had valid, legal, permitted residence in Colombia, or irregular status, meaning that they had no legal permit to stay in Colombia or that their permit had expired. A regular migration status provides access to formal employment and access to health insurance through employment or the subsidized system in Colombia.

Food security was measured using the Spanish translation of the US Department of Agriculture Food Security Survey 6-item short-form module [24]. This module includes items measuring the self-reported frequency of the following events over the past 12 months: purchased food did not last in the household and there were insufficient funds to purchase more, the participant/other adults in the household did not have money to buy more, the participant/other adults could not afford to eat balanced meals, the participant/other adults cut the size of meals or skipped meals because there was not enough money for food, the participant ate less than they felt they should because there was not enough money for food, and the participant was hungry but did not eat because there was not enough money for food. The first two items included Likert scale responses of often true, sometimes true, and never true, and the last three items were binary yes/no responses. Participants who reported that they or other adults in the household had cut the size of their meals or skipped meals in the past 12 months were asked an additional question about the frequency at which this occurred. The scores from the individual items were standardly summed to create the classifications of high or marginal food security, low food security, and very low food security [24], estimates which we have previously reported for this study [14,15].

*Statistical analysis:* Our analysis focused on patterns of food insecurity and material hardships. For the purposes of the latent class analysis and because the Food Security Survey 6-item short-form module uses a combination of binary and ordinal measures and moderate responses such as ‘sometimes true’ were much less frequent, we dichotomized all categorical food security items, classifying ‘often’ or ‘sometimes true’ to ‘yes, occurred in past 12 months’ and ‘never true’ to ‘no, did not occur’. The optional item related to the frequency of cutting/skipping meals was also excluded from the latent class analysis. The five included binary items had an internal consistency of KR20 = 0.77. Other material hardship measures included income and housing insecurity. Income was classified as follows: (1) less than minimum wage (908,526 pesos per month), (2) minimum wage (908,526 pesos), and (3) above minimum wage (>908,526 pesos). Housing insecurity was a categorical measure of the number of nights in which the participant had difficulty finding a safe place to sleep in the past 6 months. For the purposes of this analysis, housing insecurity was collapsed to none versus one or more unsafe nights in the past 6 months. In total, seven items spanning food security and material hardships were included as indicators in the latent class analysis. We did not include an indicator for medical hardship; these typically focus on access to healthcare when needed and would have reduced our analysis of the sample to people with predominantly chronic health conditions.

We used a latent class analysis, carried out through a data-driven approach, to identify underlying patterns or classes using finite mixture modeling [25] to generate classes representing different patterns of food insecurity and material hardship experiences among Venezuelan refugees and migrants in urban Colombia. We determined the optimal number of classes based on the fit indices (Table 1) and model interpretability [26]. The participants were then assigned into different classes based on their posterior probabilities of class membership. We then conducted descriptive analyses to characterize the demographics and displacement experiences for each class and compare the differences across the classes.

We fit a multivariable multinomial logistic regression model to examine the relationship between participant characteristics and identified latent class membership for food insecurity and material hardship. Class 1 served as the base category. Independent variables included items that were conceptually or previously shown to be associated with food insecurity among refugee and migrant populations. Items that were significantly associated with predicted class membership (*p* < 0.05) in the bivariate analyses were tested for inclusion in the multivariable model; those that were no longer significant after other variables were added to the model, and those that were colinear with other variables in the model were omitted from the final multivariable multinomial model for model parsimony. The final multivariable model was examined for fit using the Hosmer–Lemeshow goodness of fit test for multinomial models [27].

Finally, we used separate adjusted logistic regression models to determine the relationship between the identified classes and participants’ physical and mental health outcomes, controlling for site, age, and migration status. The health outcomes included self-rated health, classified as fair to poor vs. good to excellent [22]; symptoms of anxiety and depression, classified as normal or mild (PHQ4 score <= 5) vs. moderate or severe (PHQ4 score >= 6) [23]; report of physical, psychological, and/or sexual violence victimization in the past 12 months [28]; and laboratory-confirmed HIV and/or syphilis infection. All statistical analyses were conducted using Stata version 17 (College Station, TX, USA).

## 3. Results

**Figure 1:**
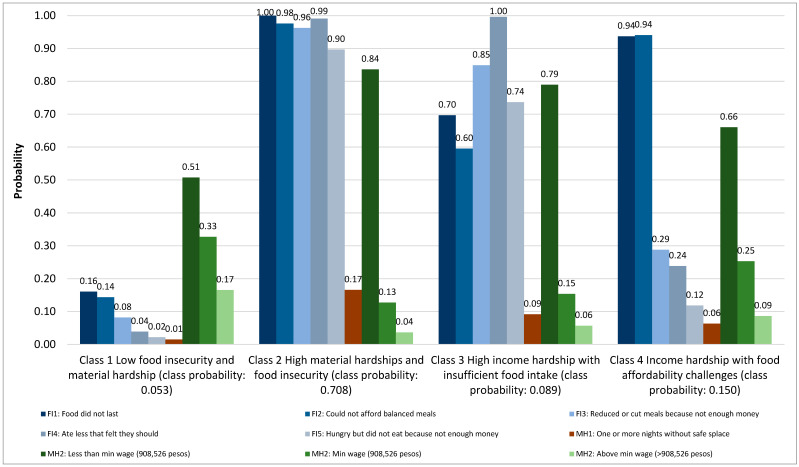
Item response probabilities of food insecurity (FI) and material hardships (MH) among Venezuelan refugees and migrants in Colombia.

A four-class model appeared to be the optimal class solution (Table 1), based on the Akaike Information Criterion (AIC) and Bayesian Information Criterion (BIC) [26]. The four classes maintained a reasonable size and interpretability, as graphed in Figure 1. The four classes appeared to represent distinct food insecurity and material hardship experiences among Venezuelan refugees and migrants in Colombia (N = 6211). Class 1 was represented by the lowest item response across all food insecurity and material hardship indicators, relative to other classes, and had the lowest probability of class membership (0.053). Class 2 reflected the highest item response across all food insecurity and material hardship indicators and had the highest probability of class membership (0.708). Class 3 appeared to represent high income hardship (probability of income below minimum wage: 0.79) with insufficient food intake (probability of eating less: 1.0; probability of reducing or skipping meals: 0.85), though other food insecurity items had probabilities that exceeded 0.60. The probability of class membership for Class 3 was 0.089. Class 4 appeared to represent income hardship (probability of income below or at minimum wage: 0.66 and 0.25, respectively) and food affordability challenges (probability that food did not last or could not afford food was 0.94 for both indicators). The probability of class membership for Class 4 was 0.150. The probability for the unsafe housing item was generally lower, though it varied by class and was as low as 0.01 in Class 1 and as high as 0.17 in Class 2.

*Participant characteristics and class membership:* Table 2 displays the sample’s characteristics and associations with posterior probabilities of class membership. The study participants had a median age of 32 years (IQR: 26–41), with two thirds of the sample comprising cisgender women. Seventy-one percent reported having an irregular migration status, though this was more commonly reported among Class 2 members than those in the other groups. Food insecurity in Venezuela was the most common motivation for migration to Colombia (52.6%), followed by job insecurity (28.0%), other reasons (12.6%), and to join family members (6.8%), though food insecurity was more commonly reported among Class 2 members. Overall, more than half (54.1%) of the participants reported that finances were their most significant challenge in Colombia, followed by food (18.7%), housing (16.9%), and other challenges (6.9%), and no challenges were reported by 3.3% of the participants. Participants in Class 2 and 3 were more likely to report that their most significant challenge was food access, while participants in Class 1 and 4 were more likely to report experiencing no challenges compared to those with membership in other classes. Almost 20% reported using humanitarian services while in Colombia, and this was more commonly reported by members of Class 2 and 3 than those in Class 1 or 4.

Corresponding adjusted prevalence ratios (aPrRs) from the multivariable multinomial regression model are also displayed on Table 2. Most characteristics were associated with class membership in the bivariate analyses. Education, chronic health condition, and the use of humanitarian resources were no longer associated with class membership after the inclusion of other variables and were omitted from the multivariable model for parsimony.

*Associations with Class 2 membership:* Residents of Barranquilla or Soledad (reference: Bogotá or Soacha; aPrR: 2.0, 95%CI: 1.5–2.5); participants aged 30 to 39 (reference: >=40 years; aPrR: 1.6, 95%CI: 1.2–2.2); cisgender women (reference: cisgender men; PrR 1.5, 95%CI: 1.2–1.9); participants with increasing numbers of dependents (aPrR: 1.1; 95%CI: 1.0–1.1); participants who were divorced, separated, or widowed (reference: never married; aPrR: 1.9, 95%CI: 1.3–2.9); participants who were unemployed (reference: full or part-time employment in formal sector; aPrR: 2.1; 95%CI: 1.5–3.0); and participants who had an irregular migration status (reference: regular; aPrR 1.4, 95%CI: 1.1–1.7) or who reported migration due to job insecurity (reference: to join family; aPrR: 2.3, 95%CI: 1.5–3.4), food insecurity (reference: to join family; aPrR: 2.4, 95%CI: 1.7–3.6), or other reasons (reference: to join family; aPrR: 2.3, 95%CI: 1.4–3.6) were independently associated with Class 2, which contained those with high material hardships and food insecurity.

*Associations with Class 3 membership:* Residents of Barranquilla or Soledad (reference: Bogotá or Soacha; aPrR: 1.6, 95%CI: 1.1–2.1); participants aged 30 to 39 (reference: >=40 years; aPrR: 1.8, 95%CI: 1.2–2.6); cisgender women (reference: cisgender men; aPrR: 1.4, 95%CI: 1.0–2.0); participants who were unemployed (reference: full or part-time employment in formal sector; aPrR: 2.3; 95%CI: 1.4–3.8); and participants who reported migration due to job insecurity (reference: to join family; aPrR: 2.3, 95%CI: 1.3–4.1), food insecurity (reference: to join family; aPrR: 2.0, 95%CI: 1.2–3.5), or other reasons (reference: to join family; aPrR: 2.3, 95%CI: 1.2–4.3) were independently more likely to be associated with Class 3, which contained those with high income hardship and insufficient food intake.

*Associations with Class 4 membership:* Participants who were aged 18 to 29 years (reference: >=40 years; aPrR: 1.6, 95%CI: 1.2–2.1) or 30 to 39 years (aPrR: 1.5, 95%CI: 1.1–2.1); divorced, separated, or widowed (reference: never married; aPrR: 1.9, 95%CI: 1.2–3.0); or who reported migration due to food insecurity (reference: to join family; aPrR: 1.6, 95%CI: 1.0–2.4) or other reasons (reference: to join family; aPrR: 1.8, 95%CI: 1.1–2.9) were independently more likely to be assigned to Class 4, which contained those with income hardship and food affordability challenges. Migration status was not associated with membership in Class 3 or Class 4.

*Associations between class membership and select health outcomes:* Table 3 displays the relationship between class membership and health indicators, including good to excellent self-rated health, self-reported symptoms of anxiety and depression, and laboratory-confirmed syphilis and/or HIV infection. Participants with membership in Classes 2, 3, and 4 were 30–60% less likely to report good to excellent health compared to those with membership in Class 1—low food insecurity and material hardship. Conversely, participants with predicted membership in these three classes had 1.8 to 4.9 times the odds of self-reported symptoms of anxiety or depression. Class 2, containing those with low food insecurity and material hardship, had the most elevated odds of anxiety and depression symptomatology, with an adjusted odds ratio of 4.9 (95%CI: 3.1–7.6), compared to those in Class 1. There was no significant association between predicted class membership and laboratory-confirmed HIV and/or syphilis infection, though there was some evidence to suggest a marginal association (direction of effect and *p* < 0.10) between Class 2 and 4 compared to those with predicted membership in Class 1. Finally, class membership was associated with recent (past 12 months) physical, sexual, and/or psychological violence victimization. The odds of recent violence victimization among the Class 2 members were five times that of the Class 1 members (aOR: 5.2, 95%CI: 2.1–12.8).

## 4. Discussion

Drawing on data from more than 6200 Venezuelan refugees and migrants from urban settings of Colombia, we previously estimated that 92% of Venezuelans were experiencing low (26.5%) or very low (65.7%) food security and material hardships in 2021–2022 [14,15]. While not directly comparable due to methodological differences, a study conducted by the World Food Programme in 2022 reported that 26% and 4% of the Colombian population were moderately food insecure and severely food insecure, respectively [29]. In this analysis, we identified four latent classes of food insecurity and material hardships: Class 1—low food insecurity and material hardship; Class 2—high food insecurity and material hardship; Class 3—high income hardship with insufficient food intake; and Class 4—income hardship with food affordability challenges. Simply put, Class 1 reflected the lowest forms of food insecurity and material hardships, while Class 2 reflected the most severe food insecurity and material hardships, and these two classes had the smallest and largest class memberships, respectively. Other investigators, through research with non-displaced populations, have proposed that measures of material hardships reflect different time horizons and economic conditions [17]. Specifically, they argue that food insecurity reflects small, short-term variations in resources, expense hardship reflects compounding short-term economic shocks, and housing insecurity reflects long-term economic constraints [17]. Thus, while food insecurity was common, it is perhaps a positive sign that we observed unsafe housing to have the lowest response probability of any material hardship measure across all latent classes. These findings may also reflect resilience through social relationships among refugees and migrants that support safe housing.

Paradoxically, consistent with prior qualitative research [20], food and job insecurity were the most common motivators for migration to Colombia, yet food insecurity and related material hardships persisted despite the duration of time for which one resided in Colombia. Further, these motivators were consistently associated with membership in all three classes with elevated food insecurity and material hardships (Classes 2 through 4). Nevertheless, it is important to consider that the material conditions of refugees and migrants change over time, and it is often expected that those migrants who manage to regularize and achieve economic integration will see an improvement in their situation. Despite the fact that we measured length of stay and migration status among the participants, the cross-sectional nature of this study does not allow us measure heterogeneous changes in food insecurity and material hardships over time. Other research has shown mixed results in which some health outcomes have improved while others have worsened for migrants and refugees in this setting [13]; thus, further research is needed to inform how to support migrants and refugees as they become established in host settings and able to regularize their status.

Legal protection in a host setting is critical to the realization of human rights, health protection, and social advancement for refugees and migrants in host settings and is equally relevant in this context [30]. Venezuelan refugees and migrants with an irregular migration status, which comprised over 70% of the population at the time, were independently more likely to have greater levels of food insecurity and material hardships (i.e., Class 2). This is likely explained by a lack of access to formal employment and other benefits afforded to those with a regular status through visas, citizenship, or other permits allowing them to stay. The findings regarding a lack of difference between the Class 3 and 4 members in terms of migration status are also noteworthy and suggest that, although a regularized status may protect against the most severe forms of food insecurity and material hardships (i.e., Class 2), Venezuelan migrants and refugees of any migration status continue to experience some patterns of food insecurity and material hardships (i.e., Classes 3 and 4). This is an important consideration in light of the Colombian government’s decision to establish the 10-year Temporary Protection Statute for Venezuelans (ETPV) [31]. The ETPV would benefit Venezuelans with an irregular status and is potentially the most significant structural intervention for this group in that it grants access to formal employment, health insurance, and other benefits once an individual has received their permit. The ETPV was established in 2021, and by December 2023, 1.86 million Venezuelans had completed all steps and received their Temporary Protection Permits [32]. Inferring from our study findings, the ETPV may help to reduce the most severe forms of food insecurity and material hardships, but additional social support and safety nets may be needed fully reduce food insecurity and material hardships among Venezuelan refugees and migrants. All of the above serves as evidence that the health of migrants in general and food security are particularly dependent on migration policy in the medium and long term [33]. While specific programs to identify and address food insecurity among migrants need to be further developed, especially for irregular migrants, it is equally important to strengthen policies including regularization, as well as the social and economic inclusion of migrants. These efforts indeed impact the social determinants of food insecurity and malnutrition.

Other characteristics, such as age, gender, city of residence, and employment status were associated with class membership and consistent with findings on Venezuelan refugees and migrants in other countries, as well as research on Colombian citizens [10,11,29]. Cisgender women were more likely than cisgender men to experience almost all classes of food insecurity and material hardships. While other research has suggested that heightened food insecurity among women may be explained by separation from one’s spouse and/or gender norms related to work and parenting [5,34], the association in our analysis persisted even after controlling for marital status, number of dependents, and employment status. The number of dependents was also independently associated with the highest levels of food insecurity and material hardships (i.e., Class 2 membership), likely reflecting the challenge of supporting and providing nutrition for more family members and suggesting greater social protection needs for large households. Finally, we found a consistently higher probability of membership in all food insecurity and material hardships (i.e., Classes 2 through 4) among Venezuelans who resided in Barranquilla or Soledad relative to those who resided in Bogotá or Soacha. Barranquilla and Soledad are known to have a lower cost of living compared to Bogotá and Soacha and, therefore, may attract Venezuelans who have greater socioeconomic vulnerabilities but may also have more limited economic opportunities. Nonetheless, this finding underscores the need to ensure that humanitarian services and other benefits are universally accessible across settings where refugees and migrants reside.

Food insecurity and material hardship class membership was associated with self-rated health, mental health symptoms, and recent violence victimization and marginally associated with infectious disease outcomes (laboratory-confirmed HIV and/or syphilis infection). There appeared to be a dose–response relationship in which membership in Class 2 (high material hardships and food insecurity) had the lowest odds of good to excellent self-rated health and had the highest odds of anxiety or depressive symptomatology, as well as recent violence victimization. These findings are consistent with global research that has demonstrated the direct links between food insecurity and material hardships with mental health, as well as indirect relationships with mental and physical health through malnutrition and delayed medical care [34,35,36]. Similarly, household food insecurity and material hardships have been associated with increased household stress that may trigger violent behavior, while survivors experiencing food insecurity and material hardships may not have the means to access safer housing or to escape abusive relationships, thus increasing the risk of violence victimization [37,38,39]. Ultimately, food insecurity has been independently associated with emergency medical visits, hospitalizations, and increased hospital expenditures [40]. National estimates suggest that at least 30% of the Colombian population also experienced food insecurity in 2023 [29]; thus, interventions that reduce and prevent material hardships and food insecurity among refugees and migrants, alongside the host community, may improve public health and reduce medical expenditures.

The findings should be considered in light of this study’s limitations. First, the cross-sectional study design limits temporal inferences. Furthermore, food insecurity and health events share common causes related to motivations for migration [41]. Therefore, these correlations should be interpreted as indicative of existing patterns but not interpreted as causal. These findings reflect the mixed migratory flows from Venezuela, in which some refugees and migrants primarily move for economic reasons, while others do so mainly for health-related reasons. Because we used a latent class analysis to examine the multidimensionality of food insecurity and material hardships, our estimates should not be interpreted as population estimates; population estimates of food insecurity and income have been previously reported [14,15]. Our design does not allow for the evaluation of the longitudinal patterns of changes in social determinants over time, though we recognize that heterogeneity within and across individuals occurs over time. This is relevant for future research, given the dynamic nature of migrants’ social conditions, especially considering that many of them regularize their status and substantially improve their material conditions over time through the economic and social inclusion that many achieve.

Finally, we do not know the impact that the COVID-19 pandemic and associated social distancing measures had on these findings. Global food insecurity increased during the pandemic [5], and Venezuelan refugees and migrants who participated in formative research reported loss of income and housing and indicated that others had returned to Venezuela early during the pandemic [20]. However, as a constantly changing social situation, there are no estimates of the numbers of Venezuelans affected, nor are there estimates of the numbers who returned to Venezuela. If housing and income loss during the pandemic were high, then it is possible that our findings regarding food insecurity and material hardships, may be more elevated than they would have been before the COVID-19 pandemic. Conversely, we may underestimate food insecurity and material hardship if some Venezuelans experienced worse conditions in Colombia and subsequently returned to Venezuela during the pandemic. Nonetheless, we do not believe that the pandemic affected the latent classes and relationships observed in this analysis.

## 5. Conclusions

Heterogeneous patterns of food insecurity and material hardships exist among Venezuelan migrants and refugees in Colombia. These are both causes of external migration and are persistent challenges faced by Venezuelan refugees and migrants in Colombia, as well as other host countries [10,11]. However, Venezuelans with an irregular migration status were more likely to experience the most severe patterns of food insecurity and material hardships. Regularization through the ETPV and other legal pathways may reduce severe concomitant patterns of food insecurity, unsafe housing, and economic vulnerabilities, though our data suggest that some food insecurity and material hardships may persist even for migrants and refugees with a regular status. Social safety nets, social protection programs, and other interventions that reduce and prevent material hardships and food insecurity among refugees and migrants, alongside the host community, may improve public health, support development, and reduce healthcare costs. In the long term, strengthening immigration policies—including regularization and social policies for migrants aimed at enhancing their social and economic inclusion—could significantly contribute to improving food security for migrants within this population.

## Figures and Tables

**Table 1:** Fit indices for potential latent classes (N = 6221).

Number of Classes	Log Likelihood	Akaike Information Criterion (AIC)	Bayesian Information Criterion (BIC)
1	−19,650.61	39,317.21	39,371.10
2	−16,776.07	33,586.15	33,700.66
3	−16,550.18	33,152.37	33,327.49
4	−16,452.71	32,975.42	33,211.17

**Table 2:** Participant characteristics and associations with predicted food insecurity and material hardship class membership among Venezuelans in urban settings of Colombia (N = 6221).

			Predicted Classes																			
	Total Sample (N = 6221)		Class 1: Low Food Insecurity and Material Hardship (n = 344)		Class 2: High Material Hardships and Food Insecurity (n = 4636)						Class 3: High Income Hardship with Insufficient Food Intake (n = 392)						Class 4: Income Hardship with Food Affordability Challenges (n = 849)					
	n	Col %	n	Col %	n	Col %	aPrR	95%CI		*p*-Value	n	Col %	aPrR	95%CI		*p*-Value	n	Col %	aPrR	95%CI		*p*-Value
**Site**																						
Bogotá and Soacha	3102	49.9	229	66.6	2128	45.9	Reference				206	52.6	Reference				539	63.5	Reference			
Barranquilla and Soledad	3119	50.1	115	33.4	2508	54.1	2.0	1.5	2.5	0.000	186	47.4	1.6	1.1	2.1	0.005	310	36.5	1.1	0.8	1.5	0.430
**Age**																						
18 to 29	2470	39.7	147	42.7	1761	38.0	1.1	0.9	1.5	0.419	153	39.0	1.2	0.8	1.7	0.358	409	48.2	1.6	1.2	2.1	0.004
30 to 39	1978	31.8	84	24.4	1536	33.1	1.6	1.2	2.2	0.001	134	34.2	1.8	1.2	2.6	0.003	224	26.4	1.5	1.1	2.1	0.025
40 and above	1773	28.5	113	32.8	1339	28.9	Reference				105	26.8	Reference				216	25.4	Reference			
Gender * (n = 6217)																						
Man	2124	34.2	162	47.4	1474	31.8	Reference				136	34.8	Reference				352	41.5	Reference			
Woman	4046	65.1	178	52.0	3129	67.5	1.5	1.2	1.9	0.001	251	64.2	1.4	1.0	2.0	0.029	488	57.5	1.1	0.9	1.5	0.384
Transgender or Nonbinary	47	0.8	2	0.6	32	0.7	1.6	0.4	6.8	0.531	4	1.0	2.2	0.4	12.3	0.378	9	1.1	1.9	0.4	9.0	0.418
Marital status * (n = 6220)																						
Never married	2287	36.8	148	43	1657	35.7	Reference				156	39.8	Reference				326	38.4	Reference			
Married or cohabitating	2991	48.1	164	47.7	2232	48.2	1.0	0.8	1.3	0.739	188	48	0.9	0.7	1.3	0.736	407	47.9	1.2	0.9	1.5	0.256
Divorced, separated, or widowed	942	15.1	32	9.3	746	16.1	1.9	1.3	2.9	0.002	48	12.2	1.3	0.8	2.2	0.312	116	13.7	1.9	1.2	3.0	0.006
Number of dependents (median, IQR) *	4	(3-5)	4	(2-5)	4	(3-5)	1.1	1.0	1.1	0.008	4	(3-5)	1.1	1.0	1.1	0.069	4	(3-5)	1.0	0.9	1.1	0.967
Highest completed education * (n = 6218)																						
No formal or primary only	1383	22.2	69	20.2	1069	23.1	-	-	-	-	91	23.2	-	-	-	-	154	18.1	-	-	-	-
Secondary	3429	55.1	192	56.1	2524	54.5	-	-	-	-	229	58.4	-	-	-	-	484	57.0	-	-	-	-
Higher or other	1406	22.6	81	23.7	1042	22.5	-	-	-	-	72	18.4	-	-	-	-	211	24.9	-	-	-	-
Employment * (n = 6219)																						
Formal, full-time, or part-time	749	12	67	19.5	487	10.5	Reference				40	10.2	Reference				155	18.3	Reference			
Unemployed	2283	36.7	88	25.7	1771	38.2	2.1	1.5	3.0	0.000	143	36.5	2.3	1.4	3.8	0.001	281	33.1	1.3	0.9	1.9	0.213
Informal	3028	48.7	176	51.3	2273	49	1.3	0.9	1.7	0.125	197	50.3	1.5	1.0	2.4	0.063	382	45	0.9	0.6	1.3	0.509
Other	159	2.6	12	3.5	104	2.2	1.0	0.5	2.0	0.892	12	3.1	1.5	0.6	3.8	0.354	31	3.7	1.0	0.5	2.1	0.991
Migration Status *																						
Regular	1779	28.6	124	36.0	1247	26.9	Reference				131	33.4	Reference				277	32.6	Reference			
Irregular	4442	71.4	220	64.0	3389	73.1	1.4	1.1	1.7	0.012	261	66.6	1.0	0.8	1.4	0.834	572	67.4	1.1	0.9	1.5	0.418
Motivation for migration *																						
To join family	422	6.8	43	12.5	276	6.0	Reference				25	6.4	Reference				78	9.2	Reference			
Job insecurity	1741	28.0	100	29.1	1293	27.9	2.3	1.5	3.4	0.000	122	31.1	2.3	1.3	4.1	0.004	226	26.6	1.4	0.9	2.2	0.157
Food insecurity	3275	52.6	155	45.1	2519	54.3	2.4	1.7	3.6	0.000	191	48.7	2.0	1.2	3.5	0.011	410	48.3	1.6	1.0	2.4	0.037
Other	783	12.6	46	13.4	548	11.8	2.3	1.4	3.6	0.000	54	13.8	2.3	1.2	4.3	0.011	135	15.9	1.8	1.1	2.9	0.030
Self-reported chronic health condition * (n = 6219)																						
No	5518	88.7	306	89.0	4076	88.0	-	-	-	-	361	92.1	-	-	-	-	775	91.3	-	-	-	-
Yes	701	11.3	38	11.0	558	12.0	-	-	-	-	31	7.9	-	-	-	-	74	8.7	-	-	-	-
Used humanitarian resources * (n = 6218)																						
No	5015	80.7	302	87.8	3686	79.5	-	-	-	-	309	78.8	-	-	-	-	718	84.7	-	-	-	-
Yes	1203	19.3	42	12.2	948	20.5	-	-	-	-	83	21.2	-	-	-	-	130	15.3	-	-	-	-
Most significant challenge in Colombia (n = 6218) *																						
Finances	3366	54.1	193	56.1	2528	54.6	-	-	-	-	197	50.3	-	-	-	-	448	52.8	-	-	-	-
Housing	1048	16.9	56	16.3	798	17.2	-	-	-	-	61	15.6	-	-	-	-	133	15.7	-	-	-	-
Food	1165	18.7	21	6.1	946	20.4	-	-	-	-	77	19.6	-	-	-	-	121	14.3	-	-	-	-
Other	432	6.9	40	11.6	257	5.5	-	-	-	-	39	9.9	-	-	-	-	96	11.3	-	-	-	-
No challenges	207	3.3	34	9.9	105	2.3	-	-	-	-	18	4.6	-	-	-	-	50	5.9	-	-	-	-

Notes: Col %: column percentages; aPrR: adjusted prevalence ratio from multivariable multinomial regression model with Class 1 as base category; 95%CI: 95% confidence interval; * differences associated with class membership in bivariate analysis based on *p* < 0.05; - indicates that no aPrR and corresponding statistics calculated were omitted from the multivariable model due to collinearity or a lack of significance once other items were included in the model. The number of analytic samples for the multivariable model was 6105 (98% of full sample) due to this study being a complete case analysis.

**Table 3:** Relationship between food insecurity and material hardship class membership and key health indicators among Venezuelan migrants and refugees in urban Colombia.

	Good to Excellent Self-Rated Health (n = 4750, 76.4%)				Self-Reported Symptoms of Anxiety or Depression (n = 1373, 22.1%)				Laboratory-Confirmed HIV or Syphilis Infection (n = 378, 6.1%)				Physical, Sexual, or Psychological Violence in Past 12 Months (n = 327; 5.3%)			
Predicted Classes	aOR	95%CI		*p*-Value	aOR	95%CI		*p*-Value	aOR	95%CI		*p*-Value	aOR	95%CI:		*p*-Value
Class 1: Low food insecurity and material hardship	Reference				Reference				Reference				Reference			
Class 2: High material hardships and food insecurity	0.4	0.3	0.6	0.000	4.9	3.1	7.6	0.000	1.6	0.9	2.9	0.085	5.2	2.1	12.8	*p* < 0.001
Class 3: High income hardship with insufficient food intake	0.5	0.3	0.7	0.000	3.7	2.2	6.1	0.000	1.5	0.7	3.0	0.256	3.4	1.3	9.5	0.017
Class 4: Income hardship with food affordability challenges	0.7	0.5	0.9	0.023	1.8	1.1	3.0	0.020	1.8	1.0	3.3	0.069	2.3	0.9	6.0	0.093

Notes: aOR: adjusted odds ratio calculated from multivariable logistic regression models. Each model was also adjusted for site, age, and migration status. 95%CI: 95% confidence interval.

## Data Availability

Data are available upon request due to privacy and ethical reasons.
